# Serum metabolic profiling of rats infected with *Clonorchis sinensis* using LC-MS/MS method

**DOI:** 10.3389/fcimb.2022.1040330

**Published:** 2023-01-06

**Authors:** Su Han, Xiaoli Zhang, Jian Ding, Xiang Li, Xueli Zhang, Xu Jiang, Shanshan Duan, Beibei Sun, Xinyi Hu, Yannan Gao

**Affiliations:** ^1^ Department of Public Health and Preventive Medicine, Wuxi School of Medicine, Jiangnan University, Wuxi, China; ^2^ Department of Parasitology, Harbin Medical University, Harbin, China; ^3^ Beijing Obstetrics and Gynecology Hospital Capital Medical University, Beijing Maternal and Child Health Care Hospital, Beijing, China; ^4^ Clinical Laboratory, Zhuhai Maternal and Child Health Hospital, Zhuhai, China; ^5^ Department of Stomatology, Laixi People’s Hospital, Qingdao, China; ^6^ Department of Graduate Studies, The Fourth Affiliated Hospital of Harbin Medical University, Harbin, China

**Keywords:** clonorchiasis, serum, metabolic pathway, untargeted metabolomics, targeted metabolomics

## Abstract

**Background:**

Clonorchiasis is an important foodborne parasitic disease. The omics-based-techniques could illuminate parasite biology and further make innovations in the research for parasitic diseases. However, knowledge about the serum metabolic profiles and related metabolic pathways in clonorchiasis is very limited.

**Methods:**

A untargeted ultra-high performance liquid tandem chromatography quadrupole time of flight mass spectrometry (UHPLC-QTOF-MS) was used to profile the serum metabolites of rats at both 4 and 8 weeks post infection (wpi) with *Clonorchis sinensis* (*C. sinensis*). Additionally, multivariate statistical analysis methods were employed to identify differential metabolites. Next, serum amino acids and phosphatidylcholines (PCs) levels were determined by targeted metabolomics analysis.

**Result:**

A total of 10530 and 6560 ions were identified in ESI+ and ESI− modes. The levels of phosphatidylcholines, glycerophosphocholine and choline were significantly changed, with the shift in lipid metabolism. Significant changes were also observed in amino acids (isoleucine, valine, leucine, threonine, glutamate and glutamine). Targeted analysis showed that BCAAs (isoleucine, valine, leucine) levels significantly increased at 4 wpi and decreased at 8 wpi; threonine was increased at 8 wpi, whereas glutamate and glutamine showed a decreasing trend at 8 wpi. Additionally, the level of 17 PCs were significantly changed in infected rats. Marked metabolic pathways were involved in clonorchiasis, including glycerophospholipid metabolism, alanine, aspartate and glutamate metabolism, histidine metabolism and pyrimidine metabolism.

**Conclusion:**

These results show that *C. sinensis* infection can cause significant changes in the rat serum metabolism, especially in amino acids and lipids. The metabolic signature together with perturbations in metabolic pathways could provide more in depth understanding of clonorchiasis and further make potential therapeutic interventions.

## Introduction

Clonorchiasis is an important food-borne and zoonotic parasitic disease caused by *Clornorchis sinensis* (*C. sinensis*) infection (Han et al., 2012; Dong and Soong, 2021). Humans get infected by ingesting raw fresh water fish and shrimp that contain metacercariae (Lun et al., 2005a). Clonorchiasis is mostly prevalent in Vietnam, Korea and China, as well as 15-20 million people are estimated to be infected (Na et al., 2020). Chronic infection with *C. sinensis* results in chronic inflammation, which causes periductal fibrosis and even hepatobiliary diseases (Qian et al., 2016). Clinical manifestations of clonorchiasis present asymptomatic, epithelial hyperplasia, periductal and hepatic fibrosis, and even cholangiocarcinoma (Keiser and Utzinger, 2005). Significantly, *C. sinensis* had been classified as a grade 1 biological carcinogenic agent (Bouvard et al., 2009). Recently, our research reported the aberrant expression of hepatic microRNA, long non-coding RNA and mRNA in clonorchiasis (Han et al., 2016; Han et al., 2021). We also found hepatocyte apoptosis and iron overload in the liver of rats with *C.sinensis* infection (Han et al., 2017). However, the molecular pathogenesis underlying clonorchiasis remains unclear. Therefore, exploring the molecular mechanism of clonorchiasis could be helpful for the development of preventive measures and targeted drugs.

Recently, several “omics” techniques, including transcriptomics, proteomics, and metabolomics, were used to explore the intricate relationship between parasites and hosts (Zhou et al., 2013; Pittman et al., 2014; Zhou et al., 2017). The omics-based techniques could illuminate parasite biology and further make innovations in the research of parasitic diseases (Yu et al., 2020). Notably, metabolomics provides a powerful tool for phenotypic biology, monitoring therapeutic effectiveness and exploring biomarkers (Zhou et al., 2017). Metabolomics has been employed to profile the metabolic alterations of parasitic diseases, including *Schistosoma japonicu*m *(S. japonicum)* and *Opisthorchis felineus* in rodent models (Kokova et al., 2020; Qian et al., 2020). Similarly, we also found the changes of spleen metabolites and regulatory pathways during clonorchiasis (Zhang et al., 2020). Metabolomics contributes to supply dynamic alteration and explore the molecular mechanisms in these diseases (Yu et al., 2020).

Considering that serum samples are easy to extract and analyze, belong to the most accessible body fluids, serum samples can reflect the dynamic changes of the metabolome of the whole organism and have been used in most metabolomic studies (Dunn et al., 2011; Li et al., 2016). For example, Huang et al. used UHPLC-MS-based metabolomics to detect serum profiling changes in *S. japonicum-*infected mice and found some decreased metabolites were closely correlated with the progression of schistosomiasis (Huang et al., 2020). Zhou et al. applied Mass-Spectrometry to investigate systemic serum metabolic changes in *T. gondii*-infected mice (Zhou et al., 2017). The metabolomic analysis of serum samples is widely used for phenotypic biology and clinical biomarker discovery in parasitic diseases (Zhou et al., 2017). However, knowledge about the serum metabolic profiles and related metabolic pathways in clonorchiasis is very limited.

In this present study, liquid chromatography tandem mass spectrometry (LC-MS/MS) based metabolomics analysis was applied to investigate the rat serum metabolic profiles in clonorchiasis. These results will offer a new insight from systematic metabolic aspect into the molecular mechanisms of host-parasite interactions.

## Materials and methods

### Ethical approval

The Medical Ethics Review Committee of Harbin Medical University reviewed and ethically approved this study. All procedures with animals were carried out based on the National Guidelines for Experimental Animal Welfare (Ministry of Science and Technology of the People’s Republic of China, 2006) and were approved by the Animal Welfare Committee of Harbin Medical University. Additionally, significant efforts to reduce animal suffering and the number of animals were made.

### Rat experiments

Metacercariae of *C. sinensis* were obtained from infected *Pseudorasbora parva* caught from the Songhuajiang River of Heilongjiang Province. The method of collecting and purifying metacercariae are showed as below. We placed the fish in an ice box at 0°C and then transported to the laboratory. Next, we washed the fish with running water, crushed the tissue with a Waring Blender, and then digested with artificial gastric juice (0.5% pepsin in 1% HCl) at 37°C for more than 12 h. At last, the digested mixture was filtered three times, passed through sieves of 1000, 300, and 106 µm. Metacercariae were isolated by centrifugation, collected and preserved at 0.1 M phosphate buffered saline (PBS, pH = 7.4) at 4°C. A total of 12 wistar rats (specific pathogen free, SPF), aged about 6-8 weeks were bought from the Harbin Medical University Laboratory Animal Center. All rats had free access to standard chow and water to acclimate to their environment for 1 week before modeling. All rats were randomly divided into two groups, infected group (n=6) was individually infected orally with 100 *C. sinensis* metacercariae, control group (n=6) was administered with 100 µL of sterile normal solution as controls. The blood samples were collected from tail vein at 4 and 8 weeks post infection (wpi) with *C. sinensis*, respectively. All animals were weighed and the food intake of rats was monitored at 4 wpi and 8 wpi.

The Kato-Katz method was used to observe whether the rats had successfully infected with *C. sinensis*. A total of 41.7 mg of feces were collected. The number of eggs per smear was converted to the eggs per gram of feces with multiplying by 24 (Hong et al., 2003). The rats were sacrificed at 8 wpi, *C. sinensis* adults were collected from bile ducts of infected rats and flushed with saline. Worm recovery rate was determined by dividing the recovered worm number by number of metacercariae infected.

### Detection of biochemical indices and inflammatory factors

The contents of aspartate aminotransferase (AST) and alanine aminotransferase (ALT) in serum were measured with commercial reagent kits (Nanjing Jiancheng Bioengineering Institute, Jiangsu, China). The experimental procedure was processed following manufacturer’s instructions. Additionally, the level of inflammatory factors (IL-1β, IL-6, IL-10 and TNF-α) were detected by Luminex 200 liquid suspension microarray and Bio-Plex Pro Rat Cytokine 1 10PLX EXP Kit. The experimental procedure briefly described as follows: 50 µL diluted mixed micro-beads was added into wells, then 50 µL standard products and samples were added to each well (diluted twice with assay buffer in advance). The plate was incubated with shaking at room temperature for 60 min and washed three times. After that, re-incubated with 25 µL of biotin-labeled detection antibody for 30 min and washed three times. After washing, the samples were re-incubated in 50 µL diluted temperature for 10 min at room temperature. Following another washed, the magnetic beads were re-suspended with 125 µL Assay Buffer. Each sample was measured in duplicate. Data were analyzed and statistics calculated with GraphPad Prism 4 software (San Diego, CA, USA).

### Preparation of serum sample and metabolite extraction

The blood samples obtained at 4 wpi and 8 wpi were centrifuged at 3,000 g for 10 min at 4°C. The serum samples were frozen at −80°C until used for metabolite extraction. The frozen serum samples (50 μL) were placed in an EP tube, and added 200 μL extract solution (acetonitrile: methanol = 1: 1) including isotopically-labelled internal standard mixture. The mixture was vortexed for 30 s, and sonicated in ice water bath for 10 min. The mixture was kept at -40 °C for 1 h, followed by centrifugation at 12000 rpm for 15 min at 4 °C. The supernatant (200 μL) was transferred to a fresh tube and then dried in a vacuum concentrator at 37 °C. The samples were dissolved in 100 μL of 50% acetonitrile, vortexed for 30 s, and sonicated on ice for 10 min. After centrifugation at 13000 rpm for 15 min at 4°C, 75 μL of supernatant was placed in a fresh glass vial for LC/MS analysis. The quality control (QC) sample was collected by Mixing an equal aliquot of the supernatants from all samples.

### Untargeted serum metabolomics analysis

LC-MS/MS analyses were carried out using a ExionLC Infinity series UHPLC System (AB Sciex). Compounds were separated by HPLC on a Waters ACQUITY UPLC BEH Amide (2.1 * 100 mm, 1.7 μm, Waters). The mobile phase consisted of solvent A (25 mmol/L ammonium acetate and 25 mmol/L ammonia hydroxide in water, pH = 9.75) and solvent B (acetonitrile). The column temperature was maintained at 25°C, and the auto-sampler temperature was 4°C. The gradient elution procedure was run as below: 0~0.5 min, 95% B; 0.5~7.0 min, 95%~65% B; 7.0~8.0 min, 65%~40% B; 8.0~9.0 min, 40% B; 9.0~9.1 min, 40%~95% B; 9.1~12.0 min, 95% B. The injection volume was 2 μL (pos) or 2 μL (neg), respectively, and the flow rate was set at 0.5 mL/min.

The TripleTOF 5600 mass spectrometry (AB Sciex) was applied to acquire MS/MS spectra based on information-dependent acquisition (IDA) in LC/MS analyses. The data acquisition software (Analyst TF 1.7, AB Sciex) automatically the complete scan survey MS data as it collects and triggers the acquisition of MS/MS spectra according to preselected criteria.

For each cycle, 12 precursor ions with the strongest intensity and greater than 100 were selected for MS/MS. The collision energy (CE) was 30 eV, and the cycle time was 0.56 s. ESI source parameters were set as below: Gas 1: 60 psi, Gas 2: 60 psi, Curtain Gas: 35 psi, Source Temperature: 600 °C, Declustering potential: 60 V, Ion Spray Voltage Floating (ISVF): 5000 V in positive modes or -4000 V in negative modes.

### Metabolite identification and multivariate statistical analysis

The raw data (.wiff) files generated by MS were converted into mzXML format using ProteoWizard. Following conversion, data was processed with R package XCMS(version 3.2) to obtain peak deconvolution, alignment and integration. Minfrac and cut off were set as 0.5 and 0.3 respectively. Metabolites were identified using an internal MS2 database (m/z and retention). Before multivariate statistical analyses, the data were normalized by total area normalization, and the raw peak area for each metabolite was divided by the total peak area of all metabolites to calculate the relative abundance. Then the data were mean-centered and scaled using UV scaling or pareto scaling. After that, the data was imported to SIMCA-P (version 13.0, Umetrics, Umea, Sweden) for multivariate statistical analyses, including principal component analysis (PCA) and orthogonal partial least-square discriminant analysis (OPLS-DA). PCA and OPLS-DA were performed to distinguish infected from control group. The quality of the models was assessed based on the value of R2 and Q2. The variable importance in the projection (VIP) value of OPLA-DA mode was used to illustrate its contribution to the classification.

Student’s t test was used to test the statistical significance differences, and *P*-values (q-value) < 0.05 was considered as statistically significant. In order to observe the distinctions in metabolic state between infected group and control group, log_2_ transformation was performed for cluster analysis. Log2 fold change (FC) was used to assess the changes in the abundance of metabolites between different groups. In this study, based on other studies (Zhou et al., 2017; Liu et al., 2019; Zheng et al., 2019), VIP > 1, *P <*0.05 and FC ≥ 1.2 and < 0.833 were used to select differential metabolites. Heatmaps were used to depict the relatively disorder and unbalanced metabolic profile of *C. sinensis*-infected rats compared to control rats. On the basic of abundance of differentially expressed metabolites data (log2-scaled), MultiExperiment Viewer (MeV) v. 4.9 software (http://mev.tm4.org/) was used to generate heatmaps. Metabolite pathways of the differentially expressed metabolites in both ion modes were analyzed using KEGG database (http://www.genome.jp/kegg/) and MetaboAnalyst 3.0. The pathways influenced after infection were obtained, based on the *p*-values from the pathway enrichment analysis (y-axis) and pathway impact values from pathway topology analysis (x-axis).

### Targeted serum amino acids profiles analysis

A total of 25 amino acids in serum samples were determined by targeted UPLC-MS/MS method, due to their significant differential abundance from the untargeted metabolomics analysis. Almost 50 μL serum sample was employed for metabolite extraction, then 250 μL acetonitrile/methanol/formic acid (74.9:24.9:0.2,v/v/v) was added to the serum, which contained additional stable isotope labeled internal standards of valine-d8 and phenylalanine-d8. The mixture was vortexed for 1 min and kept at room temperature for 10 min, and then centrifuged at 14,000 g for 10 min at 4 °C. After that, the supernatant was placed into the sampling vial pending UPLC-MS/MS analysis.

The targeted analysis was performed on a Waters ACQUITY UPLC system (Waters Corporation, Milford, MA, USA) coupled with a Waters Xevo TQD Mass Spectrometer (Waters Corporation, Manchester, UK). The separation was performed on ACQUITY UPLCTM HILIC column (100 mm × 2.1 mm i.d., 1.7 μm; Waters Corporation, Milford, MA, USA). The UHPLC/MS-MS program was performed as follows. Briefly, the sample injection volume was 2 μL, and the flow rate was 300 μL/min. Substances were recovered from the column by gradient elution (Liu et al., 2015). ESI and Selected Reaction Monitoring (SRM) scans in the positive ion mode were used for mass spectrometric detection. In each transition, cone voltage and collision energies were optimized, with the ion spray voltage 3.2 kV, and the source temperature 150 °C. Internal standard peak areas (valine-d8 and phenylalanine-d8) were monitored for quality control and individual samples with peak areas differing from the group mean by more than two standard deviations were reanalyzed (Additional Files: **Table S1**). MarkerLynx Application Manager software (Version 4.1; Waters Corporation, Milford, MA, USA) was used for automated peak integration. The metabolite peaks were manually checked for the quality of integration.

### Targeted serum lipidomics (phosphatidylcholines) analysis

For phosphatidylcholines analysis, 10 μL serum sample was mixed with 190 μL water and 480 μL extract solution (MTBE: MeOH = 5: 1) containing internal standards. The mixture was vortexed for 60s, and sonicated for 10 min. Then the mixtures were centrifuged at 3000 rpm for 15 min at 4°C. A total of 250 μL supernatant was transferred to a fresh tube. The remaining sample was mixed with 250 μL MTBE, followed by vortex, sonication and centrifugation. After that, another 250 μL supernatant was taken out and repeated the above steps once. Subsequently, supernatants were collected and combined and dried in a vacuum concentrator at 37 °C. The dried samples were reconstituted with 100 μL of resuspension buffer (DCM: MeOH: H2O = 60: 30: 4.5), then vortexed (30 s), sonicated (10 min), and centrifuged (12000 rpm for 15 min at 4°C). Finally, 30 μL supernatant was taken out for LC/MS analysis using a UHPLC system (1290, Agilent Technologies) with a Phenomen Kinetex C18 column (2.1 × 100 mm, 1.7 μm) coupled to TripleTOF 6600 mass spectrometry (AB Sciex). The quality control (QC) sample was prepared by mixing the same volume of supernatants from all samples.

Targeted serum lipidomics analysis was performed by a SCIEX ExionLC series UHPLC System (ACQUITY UPLC HSS T3, 1.8 μm, 2.1 × 100 mm) coupled with AB Sciex Trap 6500+ mass spectrometer (AB Sciex). The mobile phase consisted of solvent A (40% water, 60% acetonitrile, and 10 mmol/L ammonium formate) and solvent B (10% acetonitrile, 90% isopropanol, and 10 mmol/L ammonium formate). The column temperature was maintained at 45°C, the auto-sampler temperature was 6°C, and the injection volume was 2 μL. Ion source parameters were set as below: ISVF: +5500/-4500 V, Curtain Gas: 40 psi, Temperature: 350°C, Ion Source Gas1: 50 psi, Ion Source Gas2: 50 psi, DP: ± 80V. The target compounds were quantitatively analyzed by Biobud-v2.1.4.1 software. Based on the peak area and the actual concentration of the same lipid internal standard (IS), the absolute concentration of lipids was calculated.

### Statistical analysis

For the data obtained by targeted analysis, the results were expressed as means ± standard deviation (SD), SPSS Statistics software version 22 (IBM, Armonk, NY, USA) was used for statistical analysis, and Prism version 8.0.1 (GraphPad Software, San Diego, CA) was used to generate box-plots (*P <0.05, **P <0.01). To identify the important metabolites of *C. sinensis* infection, the area under the curve was assessed in receiver operating characteristic analyses.

## Results

### Animal infection and assessment of biochemistry and inflammatory factors

Infection was identified by the detection of *C. sinensis* eggs in rat feces from all infected rats (**Figure S1**). Rats were sacrificed at 8 wpi, the mean worm recovery rate in the infected group was over 50%. No worms were recovered from the control group. At 4 wpi, the food intake of rats in infection group was 8.64 ± 0.39 g, the control group was 8.99 ± 0.48 g; at 8 wpi, the food intake of rats in infection group was 24.02 ± 0.36 g; the control group was 25.30 ± 0.95 g. There was no significant difference between two groups at the two time points, respectively. Additionally, the mean body weight did not significantly differ between infected (4 wpi 294.64 ± 12.09 g and 8 wpi 395.67 ± 13.83 g) and control rats (4 con 298.5 ± 11.95 g and 8 con 403.03 ± 13.56 g), respectively. As shown in **Figure S2**, ALT and AST increased both at 4 wpi and 8 wpi in the infected group (*P <*0.05). The level of IL-6, IL-10 and TNF-α were significantly increased both at 4 wpi and 8 wpi (*P <*0.05), while there was no significant change in IL-1β at two time points (*P >* 0.05).

### Metabolic profiles of serum after *C. sinensis* infection

**Figure 1:**
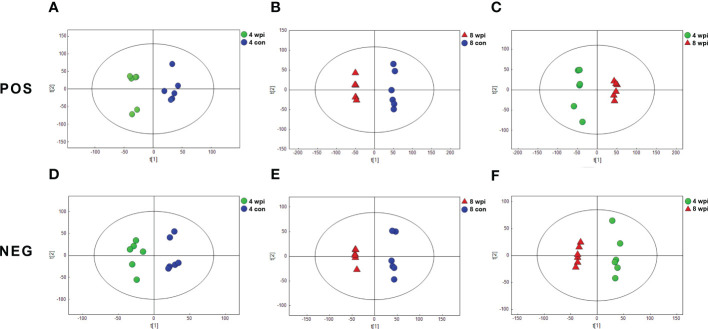
Orthogonal partial least-squares discriminant analysis (OPLS-DA) score plots showing the separation between *C. sinensis*-infected and control groups at 4 and 8 wpi in ESI+ mode **(A-C)** and ESI− mode **(D-F)**. The ellipses enclose the 95% confidence intervals estimated by the sample means and covariances of each group.

We characterized serum metabolomic changes in *C. sinensis*-infected and control groups using UHPLC-QTOF-MS. A total of 10530 and 6560 ions were identified in the positive electrospray ionization (ESI+) mode and negative electrospray ionization (ESI−) mode, respectively (Additional Files: **Table S2** and **S3**). Next, PCA score plots were first employed to depict the clustering behavior of serum metabolic profiles in different infection times. Although the PCA score plots showed a clear separation at 8 wpi, it could not clearly separate the 4 wpi group from the control (**Figure S3**). Next, the OPLS-DA analysis was performed and showed good discrimination among different infected rats groups in both ESI+ and ESI− modes (**Figure 1**). Additionally, the PCA and OPLS-DA model parameters are present in **Table S4**. These results suggest that the metabolic patterns of *C. sinensis*-infected rats are different from control rats.

### Differential metabolites in serum following *C. sinensis* infection

**Figure 2:**
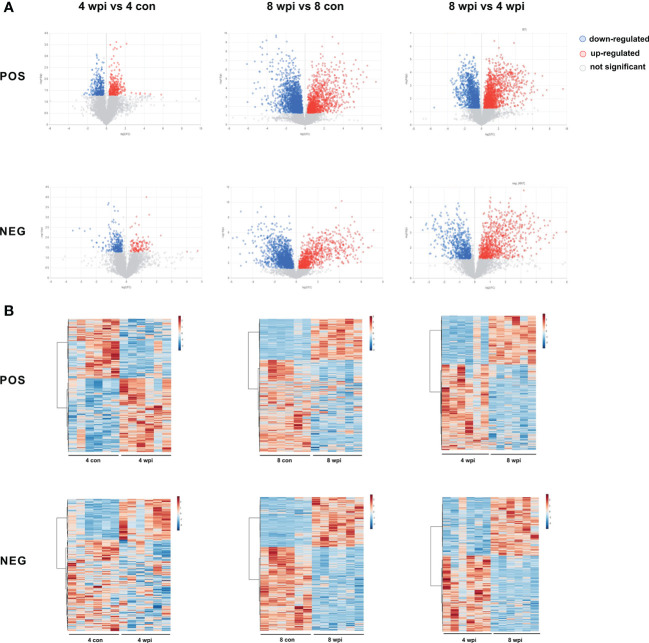
Differential metabolites were identified among different groups in ESI+ and ESI− modes. **(A)** Volcano plots showed significant differences among different groups. Each point in the map represents a metabolite. The size of the scatter represents the VIP value of the OPLS-DA model, and the larger the scatter, the larger the VIP value. (Red plot, up-regulated; Blue plot, down-regulated; Black plot, non-significant). **(B)** Heat maps representing the significantly changed metabolites between infected groups and the corresponding control groups. Each row represents data for a specific metabolite, and each column represents a rat (*C. sinensis-*infected or healthy control). Different colors correspond to the different intensity levels of metabolites. Red and blue colors represent increased and decreased levels of metabolites, respectively.

Based on the VIP > 1, *P <*0.05 and FC ≥ 1.2 and < 0.833, 522 and 3190 metabolites of serum were significantly altered at 4 and 8 wpi in the ESI+ mode, respectively. In addition, a total of 364 and 2332 metabolites were altered at 4 and 8 wpi in the ESI− mode, respectively. The volcano and heat map plots of these metabolites are present in **Figure 2**. As shown in heat map, the serum metalites of *C.sinensis*-infected rats significantly deviated from corresponding controls in ESI+ and ESI− mode. Meanwhile, the concentration of differential metabolites from 8 wpi group changed more significantly than those from 4 wpi group.

After removing the unannotated metabolites, differential metabolites in each group are shown in **Table S5**. The common and unique differential metabolites among four groups are shown by Venn diagrams (**Figure S4**). A total of 10 different metabolites were shared in the two infected groups in ESI+ mode, while only 5 metabolites were shared in ESI− mode. Both in the ESI+ mode and ESI− mode, the number of differential metabolites identified between the 8 wpi and 8 con group was larger. These results showed that the number of differential metabolites increased during the infection progresses.

### Amino acids profiles detected by targeted metabolomics

**Figure 3:**
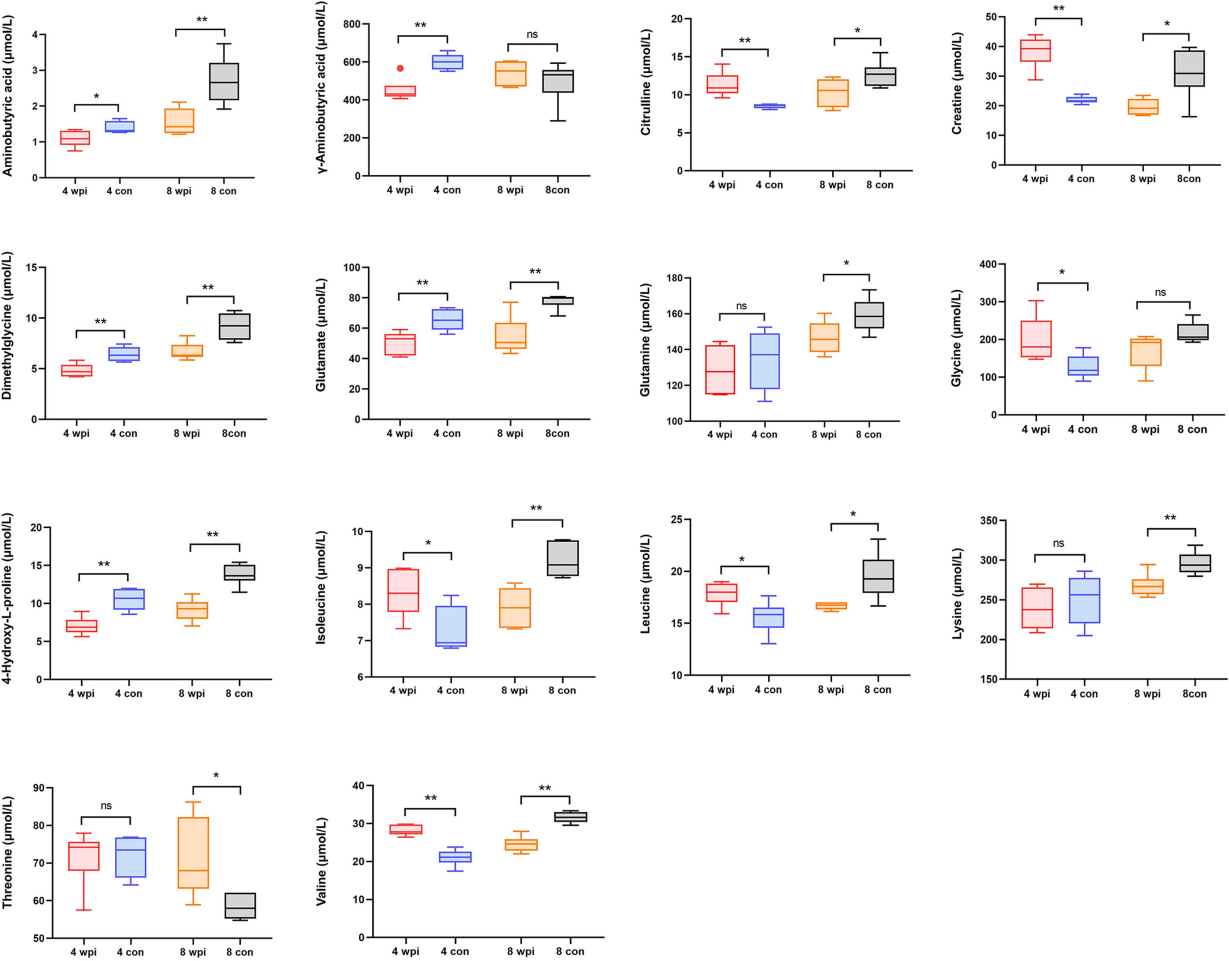
The concentrations of 14 significantly changed amino acids in serum of different groups. *P < 0.05, **P < 0.01, *t*-test. ns, no significant change.

**Figure 4:**
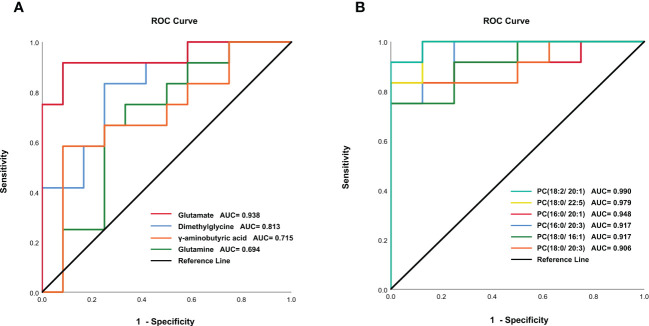
ROC analysis of amino acids metabolites **(A)** and phosphatidylcholines **(B)** in infected and control groups.

In order to validate the untargeted metabolomics analysis, 25 amino acids in serum samples were further quantitatively analyzed by targeted UPLC-MS/MS. Among them, 14 amino acids were significantly changed during *C. sinensis* infection (**Figure 3**). For example, aminobutyric acid, dimethylglycine, glutamate, and 4-Hydroxy-L-proline decreased at 4 wpi and 8 wpi. The branched-chain amino acids (BCAAs, including isoleucine, leucine and valine), citrulline and creatine increased at 4 wpi and decreased at 8 wpi. We compared the results between targeted and untargeted amino acids in **Table S6**. It was shown that glutamate and glutamine decreased at 8 wpi, and threonine increased at 8 wpi in both methods. Then metabolic pathway analysis of these amino acid metabolites was subsequently performed (Additional Files: **Table S7**). Based on *P* value < 0.05 and impact value > 0.1 (Wang et al., 2021), the main enrichment pathways included glycine, serine and threonine metabolism, arginine biosynthesis, D-glutamine and D-glutamate, glyoxylate and dicarboxylate metabolism, glutathione metabolism, and alanine, aspartate and glutamate metabolism. Additionally, the ROC analysis showed the AUC values for glutamate, dimethylglycine, γ-aminobutyric acid and glutamine of 0.938, 0.813, 0.715 and 0.694, respectively (**Figure 4A**).

### Quantification of phosphatidylcholines by targeted metabolomics

**Figure 5:**
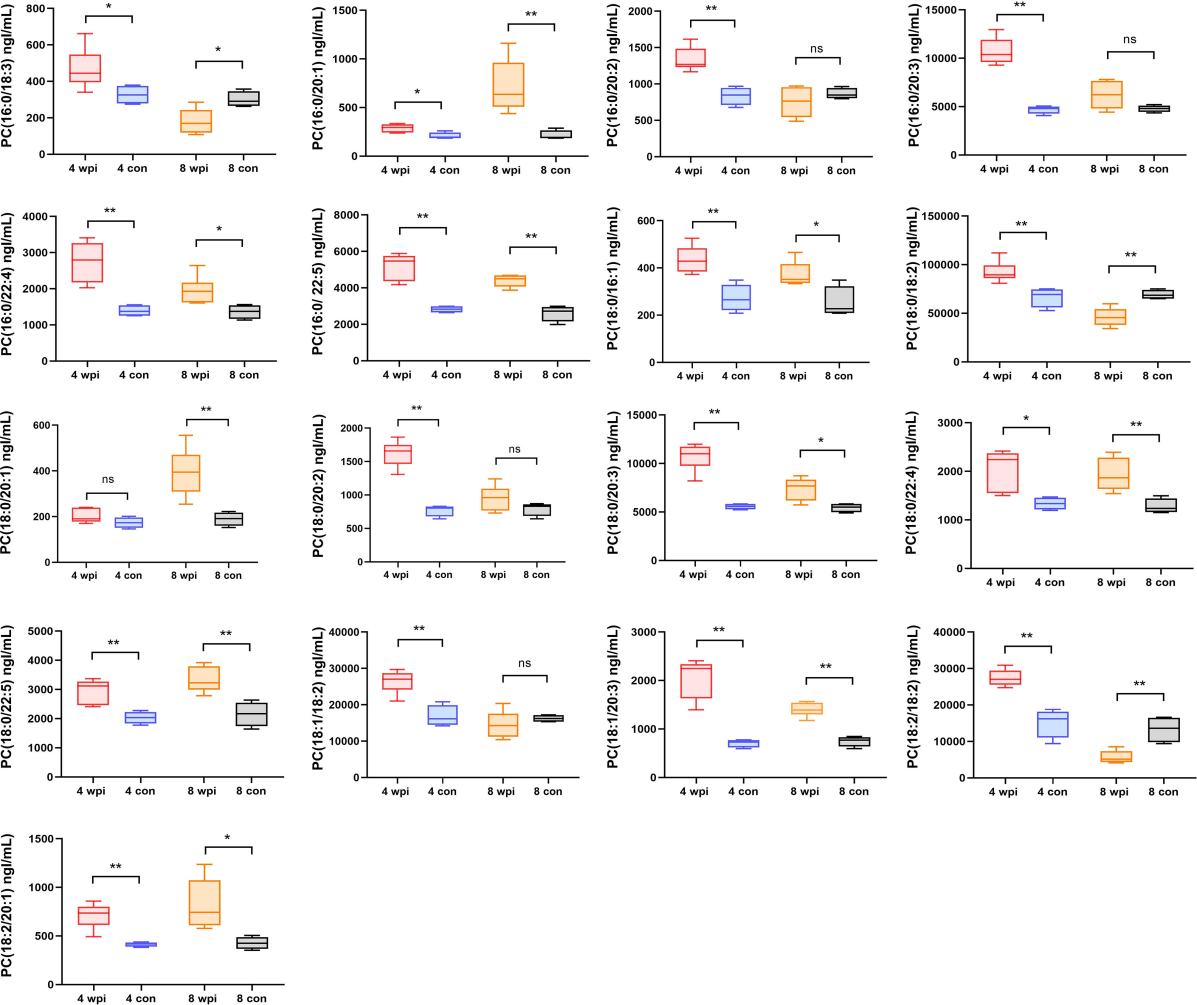
The concentrations of 17 phosphatidylcholines in serum of different groups. *P < 0.05, **P < 0.01, *t*-test. ns, no significant change.

Some lipids were also identified as the differential metabolites in untargeted metabolomics. Thus, we further analyzed the changes in the concentration of phosphatidylcholines (PCs) in serum. A total of 40 PCs were identified and 17 PCs were found differentially expressed at 4 wpi and 8 wpi (**Figure 5**). The levels of PC(16:0/20:1), PC(16:0/22:4), PC(16:0/22:5), PC(18:0/16:1), PC(18:0/20:3), PC(18:0/22:4), PC(18:0/22:5), PC(18:1/20:3), and PC(18:2/20:1) showed increased at two time points, whereas the levels of PC(16:0/18:3), PC(18:0/18:2) and PC(18:2/18:2) were increased at 4 wpi, but decreased at 8 wpi. Moreover, PC(16:0/20:2), PC(16:0/20:3), PC(18:0/20:2), and PC(18:1/18:2) showed increased only at 4 wpi, while PC(18:0/20:1) increased only at 8 wpi. According to AUC analysis, PC(18:2/20:1), PC(18:0/22:5), PC(16:0/20:1), PC(16:0/20:3), PC(18:0/16:1) and PC(18:0/20:3) had high AUC values (AUC > 0.7), which indicated that these PCs had good predictive ability for disease (**Figure 4B**).

### Metabolic pathways affected by *C.sinensis* infection

**Figure 6:**
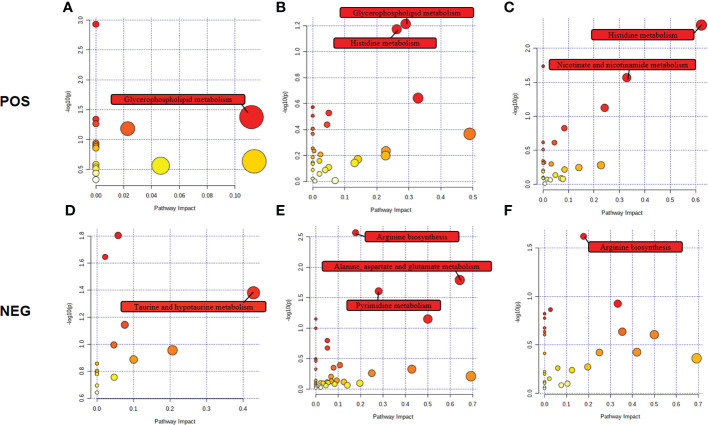
Pathway analysis of the identified differential metabolites in ESI+ mode and ESI− mode. Plots depict the pathway impacts of the key metabolites (x-axis) and the computed metabolic pathway as a function of –log (*P*) (y-axis) that different among 4 wpi vs. 4 con **(A, D)**, 8 wpi vs. 8 con **(B, E)**, and 8 wpi vs. 4 wpi **(C, F)**.

The biological metabolic pathways of the differentially expressed metabolites were determined by enrichment analysis using KEGG annotation and MetaboAnalyst. Based on *P* value < 0.05 and impact value > 0.1, the most significant differential metabolic pathways at 4 wpi were glycerophospholipid metabolism and taurine and hypotaurine metabolism; while, glycerophospholipid metabolism, histidine metabolism, arginine biosynthesis, alanine, aspartate and glutamate metabolism, and pyrimidine metabolism were significantly changed at 8 wpi, as shown in **Figure 6** and **Table S8**.

**Figure 7:**
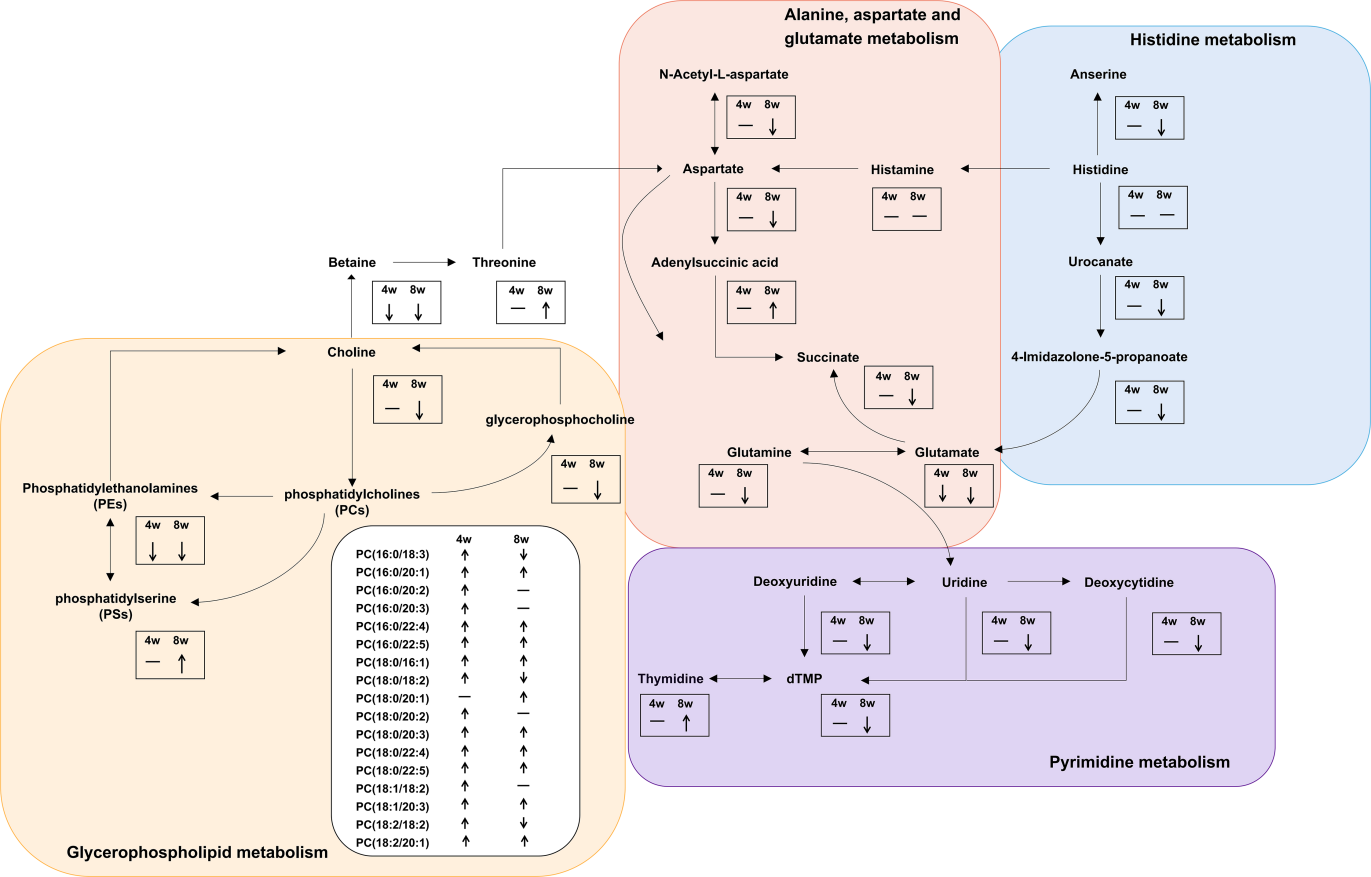
Integrated metabolic networks of significantly altered metabolic pathways during *C. sinensis* infection. The black arrows indicate up-regulation and down-regulation. Horizontal lines indicate that the metabolites are no changed.

We further made the pathway analysis of up-regulated and down-regulated metabolites (Additional Files: **Table S9**), respectively. At 4 wpi, up-regulated metabolites were enriched in glycerophospholipid metabolism and taurine and hypotaurine metabolism, while at 8 wpi, up-regulated metabolites were enriched in fructose and mannose metabolism. Down-regulated metabolites were enriched in glycerophospholipid metabolism at 4 wpi, and down-regulated metabolites were enriched in arginine metabolism, histidine metabolism, D-glutamine and D-glutamate metabolism, nicotinate and nicotinamide metabolism, pyrimidine metabolism, alanine, aspartate and glutamate metabolism, glycerophospholipid metabolism at 8 wpi. It seems that both up-regulated and down-regulated metabolites are enriched in glycerophospholipid metabolism. Finally, alanine, aspartate and glutamate metabolism, histidine metabolism, glycerophospholipid metabolism, and pyrimidine metabolism were used to construct integrated metabolic networks (**Figure 7**).

## Discussion

In this study, the serum metabolism of *C. sinensis-*infected rats at 4 and 8 wpi was analyzed by untargeted and targeted metabolomics. Because *C. sinensis* adults develop matured and start producing eggs at 4 wpi (Lun et al., 2005b), it could cause obvious inflammatory cell infiltration, fibrocyte accumulation, hepatocyte apoptosis and serious collagen deposition from 4 wpi (Zhang et al., 2008; Xu et al., 2016; Han et al., 2017). Thus, based on these important features of the worm growth, disease progress and pathologic changes, 4 and 8 wpi were selected for metabolomics analysis. Multivariate analyses demonstrated significant separation between *C. sinensis-*infected and control rats at two time points in ESI+ mode and ESI− mode, respectively. Most of these metabolites belonged to amino acids, lipids, and products of amino acid metabolism. These results suggested systemic serum metabolic perturbations in clonorchiasis.

Notably, our study observed that *C. sinensis* infection could cause significant perturbation in some amino acids. Similarly, the alteration of amino acid metabolism was observed in mice serum infected with *S. japonicum* (Huang et al., 2020). And serum amino acids levels were also found abnormal in nonalcoholic fatty liver disease patients and alcoholic liver disease rat models (Shi et al., 2020; Wang et al., 2022). The liver could synthesize a variety of amino acids, including glutamate and glutamine and plays a critical role in amino acids metabolism (Hou et al., 2020). Liver injury could cause the disturbance of serum amino acid metabolism. As basic metabolites and metabolic regulators, amino acids play significant roles in the synthesis of many cytokines and/or antibodies (Li et al., 2007), and are also involved in important metabolic pathways for growth, reproduction and immunity (Meijer, 2003; Kim and Wu, 2009; Wu, 2009). Previous studies also reported that amino acids provided important energy source for *C. sinensis* adults (Li et al., 2020). Thus, amino acid profiles might help elucidate the impact and underlying mechanisms of clonorchiasis.

The BCAAs including isoleucine, valine and leucine were found significantly increased at 4 wpi, and decreased at 8 wpi in targeted analysis (*P* < 0.05). High concentrations of BCAAs are associated with oxidative stress and inflammation under different pathological conditions (Zhenyukh et al., 2017). Elevated BCAAs levels also can activate the NF-κB signaling pathway, promoting the release of pro-inflammatory molecules, such as IL-6 and TNF-α (Zhenyukh et al., 2017). Meanwhile, the level of IL-6 and TNF-α in serum of the infected group were also identified significantly increased in our study. Increased levels of IL-6 and TNF-α may lead to liver inflammation and fibrosis (Yan et al., 2015; Higashi et al., 2017; Kang et al., 2020). Except that, during the disease progresses, BCAAs levels showed down trend at 8 wpi, which might be related to the increased utilization of these animo acids. Besides, BCAAs are important in supporting immune cell function, the deficiency of BCAAs, especially isoleucine, could impair the innate immune function in cells or organisms (Powell et al., 2012). Altogether, the changes of BCAAs may contribute to the inflammation and oxidative stress during clonorchiasis.

Threonine was another differentially expressed metabolite, increased at 8 wpi in untargeted and targeted metabolomics. Similarly, increased level of threonine was also found in *C. sinensis*-infected bile (Dalton et al., 2020). Threonine is an important component of intestinal mucin and plasma g-globulin in animals (Li et al., 2007). Therefore, the increased threonine may contribute to the maintenance of host intestinal mucosal integrity and barrier function after *C. sinensis* infection.

Both untargeted and targeted metabolomics detected glutamate and glutamine significantly decreased in infected group at 8 wpi, this is similar to previous research on serum metabolites profiles of *C. sinensis*-infected rabbits (Qiu et al., 2022). Glutamate is obtained from the catabolism of other amino acids, such as BCAAs (Cruzat et al., 2018; Holecek, 2018). Moreover, glutamate, as an immediate precursor for the synthesis of glutathione, is an important component of defense against oxidative stress (Amores-Sanchez and Medina, 1999; Johnson et al., 2003). Except that, glutamate is also an important immunomodulatory, and several glutamate receptors have been reported to be expressed on immune system cells (Pacheco et al., 2007). Therefore, it is suggested that glutamate levels may be associated with host antioxidant and immune responses during clonorchiasis. Furthermore, glutamine can be synthesized from glutamate by glutamine synthetase (Tan et al., 2017). During infection and/or high catabolism, the increased demand use for glutamine by immune system cells and other tissues (liver) (Cruzat et al., 2018), which might lead to a decrease in serum glutamine level during *C. sinensis* infection. However, it is unclear to what extent the observed changes in the abundance of these amino acids metabolites may be the result of loss of appetite, transient local inflammation, or other processes in the host, so the specific functional mechanisms of these amino acids metabolites in clonorchiasis require further investigation.

Meanwhile, this study reported that phosphatidylcholines were significantly altered in the infected group, suggesting that the glycerophospholipid metabolism was perturbed after *C. sinensis* infection. Although untargeted metabolomics could detect some lipids, there are still some limitations in the extraction and determination of lipids. Thus more PCs were further determined by targeted analysis. Lipids are important in the life cycle of the parasite and the promotion of membrane fusion in the host (Huang et al., 2020). PCs are the most abundant phospholipids in eukaryotic membranes and the main component of the tegumental outer-surface of *Schistosomain* (Van Hellemond et al., 2006). The formation of tegument plays a critical role in the uptake of nutrients and immune evasion by the parasite (Fripp, 1967; Uglem and Read, 1975; Xu and Caulfield, 1992; Van Hellemond et al., 2006). PCs also promote the proliferation and growth of cancer cells (Fagone and Jackowski, 2013; Ridgway, 2013). Elevated PCs levels have been observed in primary sclerosing cholangitis (PSC) and cholangiocarcinoma (Banales et al., 2019). Based on the previous studies, we speculate that abnormal PCs levels during clonorchiasis not only regulate the host response and parasite survival, but also is associated with the development of CCA. Additionally, dysregulation of lipids has also been found in experimental models of liver diseases in rat or mice. For example, Li et al. showed that serum levels of PCs, lysophosphatidylcholines (LPCs), and lysophosphatidylethanolamines (LPEs) were significantly increased in liver injury and hepatocellular carcinoma (Li et al., 2011). And the changes in the levels of PCs and LPCs were also detected during liver fibrosis in rats (Zhang et al., 2016). Therefore, dysregulation of lipids play important roles in both the development and the progression of liver diseases.

In addition, we also found the metabolites associated with pyrimidine metabolism were increased in the infected rats. Pyrimidine metabolism was involved in DNA and RNA biosynthesis (Tian et al., 2018; Zhao et al., 2018). The changes in pyrimidine metabolism might suggest the potential effects on DNA and RNA biosynthesis caused by *C. sinensis* infection.

Overall, we successfully identified the alteration of serum metabolome in *C. sinensis*-infected rats. These data may provide another layer of information about the molecular mechanisms of clonorchiasis. There were some potential limitations of this study. Firstly, our sample size was small, the changes in serum metabolic profiling needed to be validated by large scale samples. And further analysis of dysregulated metabolism in humans is important to estimate the specificity of the altered metabolites in humans. Second, in argeted lipidomic analysis, we only concentrated on phosphatidylcholines, while other metabolites, such as LPCs, LPEs, phosphatidylethanolamines (PEs) and phosphatidylserines (PSs), were also involved in glycerophospholipid metabolism, future research needs to explore the relationship between these lipids and the pathogenesis mechanism of clonorchiasis.

## Conclusion

In conclusion, based on LC-MS/MS-based metabolomics, we found that differential serum metabolites including amino acids (isoleucine, valine, leucine, threonine, glutamate and glutamine) and lipids (phosphatidylcholines) changed significantly in *C. sinensis*-infected rats. Some altered metabolic pathways were involved in the pathogenesis of clonorchiasis, such as glycerophospholipid metabolism, alanine, aspartate and glutamate metabolism, histidine metabolism and pyrimidine metabolism. The dysregulated metabolites, together with perturbations in metabolic pathways may provide new insights into the mechanistic understanding of pathogenesis and potential therapeutic interventions for clonorchiasis.

## Data availability statement

The raw data supporting the conclusions of this article will be made available by the authors, without undue reservation.

## Ethics statement

The animal study was reviewed and approved by Medical Ethics Review Committee of Harbin Medical University.

## Author contributions

SH and Xi-LZ conceived and designed the experiments. SH, Xi-LZ, JD, XL, Xu-LZ and XJ performed the experiments and analyzed the data. SD, BS, XH and YG contributed to reagents, materials, and analysis tools. SH, Xi-LZ and JD wrote the paper. All authors edited the manuscript, read, and approved the final version of the manuscript. All authors contributed to the article and approved the submitted version.

## Supplementary material

Supplementary Figure 1
*C. sinensis* eggs in feces of infected rats.

Supplementary Figure 2 The level of serum ALT, AST and inflammatory factors in rat infected with *Clonorchis sinensis*. ALT, alanine aminotransferase; AST, aspartate transaminase; 4 wpi, 4 weeks post infection; 4 con, 4 weeks control; 8 wpi, 8 weeks post infection; 8 con, 8 weeks control. **P*<0.05, ***P*<0.01, ****P*<0.001.

Supplementary Figure 3 PCA score scatter plots of metabolites obtained from UHPLC-QTOF-MS in ESI+ mode (A-C) and ESI− mode (D-F). Each color represents a group, and each point represents a sample. 4 wpi, 4 weeks post infection; 4 con, 4 weeks control; 8 wpi, 8 weeks post infection; 8 con, 8 weeks control.

Supplementary Figure 4 Venn diagrams displaying (comparatively) the differentially expressed metabolites. Differential metabolites across comparison groups showing unique and common metabolites in ESI+ mode (A) and ESI− mode (B).
