# Supplementary figures and images for: Serum metabolic profiling of rats infected with Clonorchis sinensis using LC-MS/MS method
Source: Front Cell Infect Microbiol. 2023 Jan 6;12:1040330. doi: 10.3389/fcimb.2022.1040330 (PMC9852996; doi:10.3389/fcimb.2022.1040330)

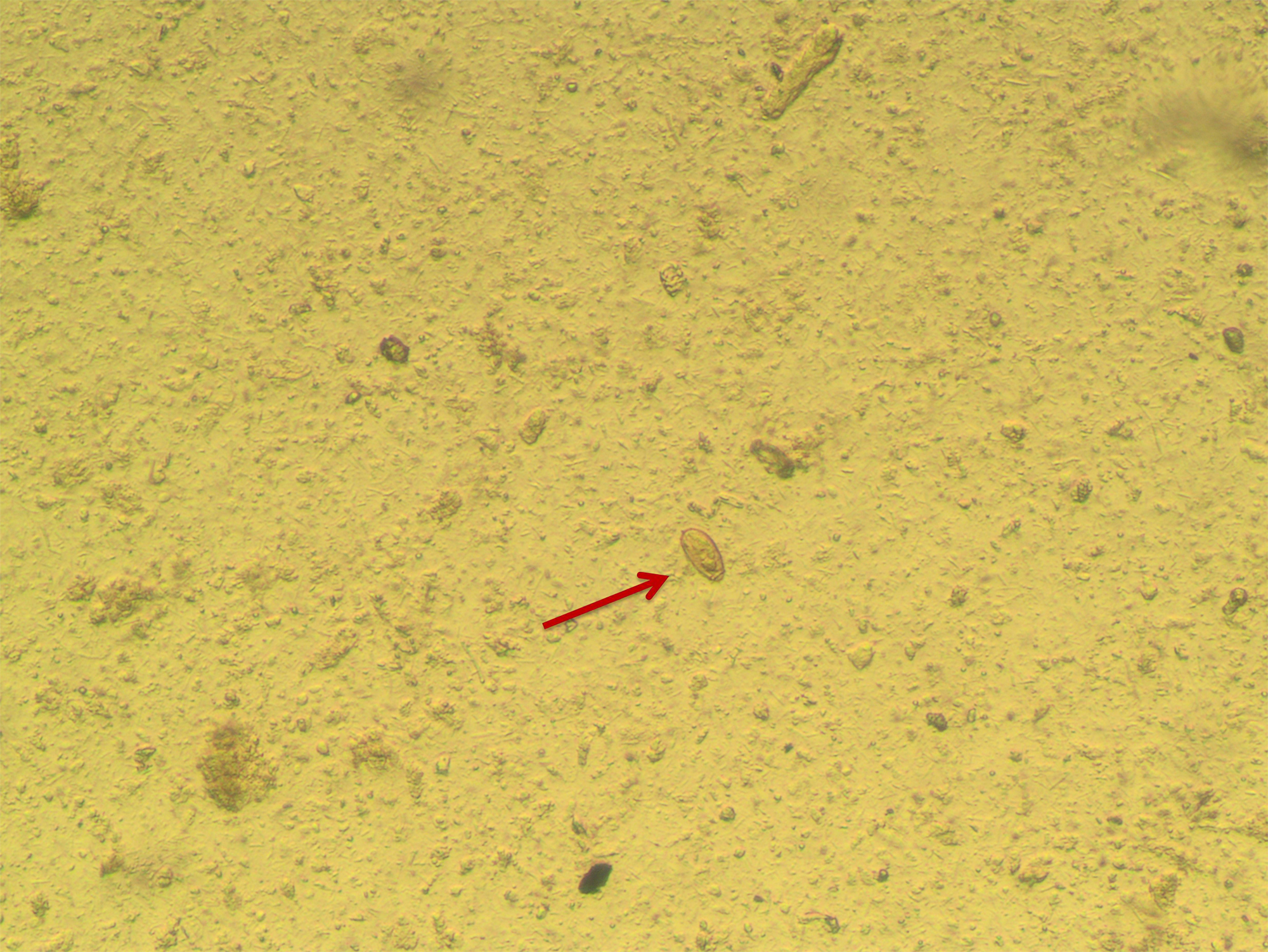

Supplement: Supplementary Figure 1 — C. sinensis eggs in feces of infected rats. [file Image_1.tif]

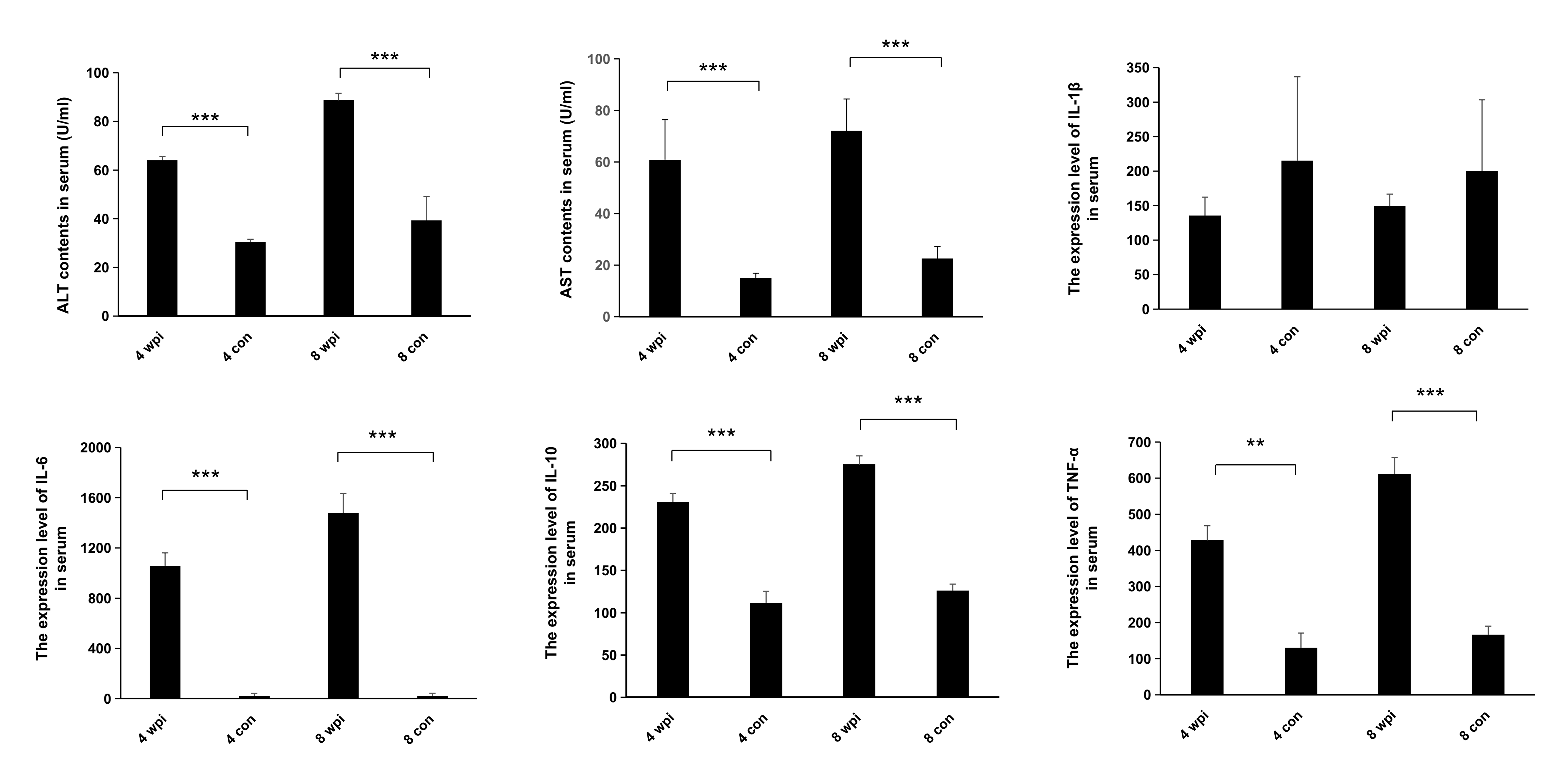

Supplement: Supplementary Figure 2 — The level of serum ALT, AST and inflammatory factors in rat infected with Clonorchis sinensis. ALT, alanine aminotransferase; AST, aspartate transaminase; 4 wpi, 4 weeks post infection; 4 con, 4 weeks control; 8 wpi, 8 weeks post infection; 8 con, 8 weeks control. *P<0.05, **P<0.01, ***P<0.001. [file Image_2.tif]

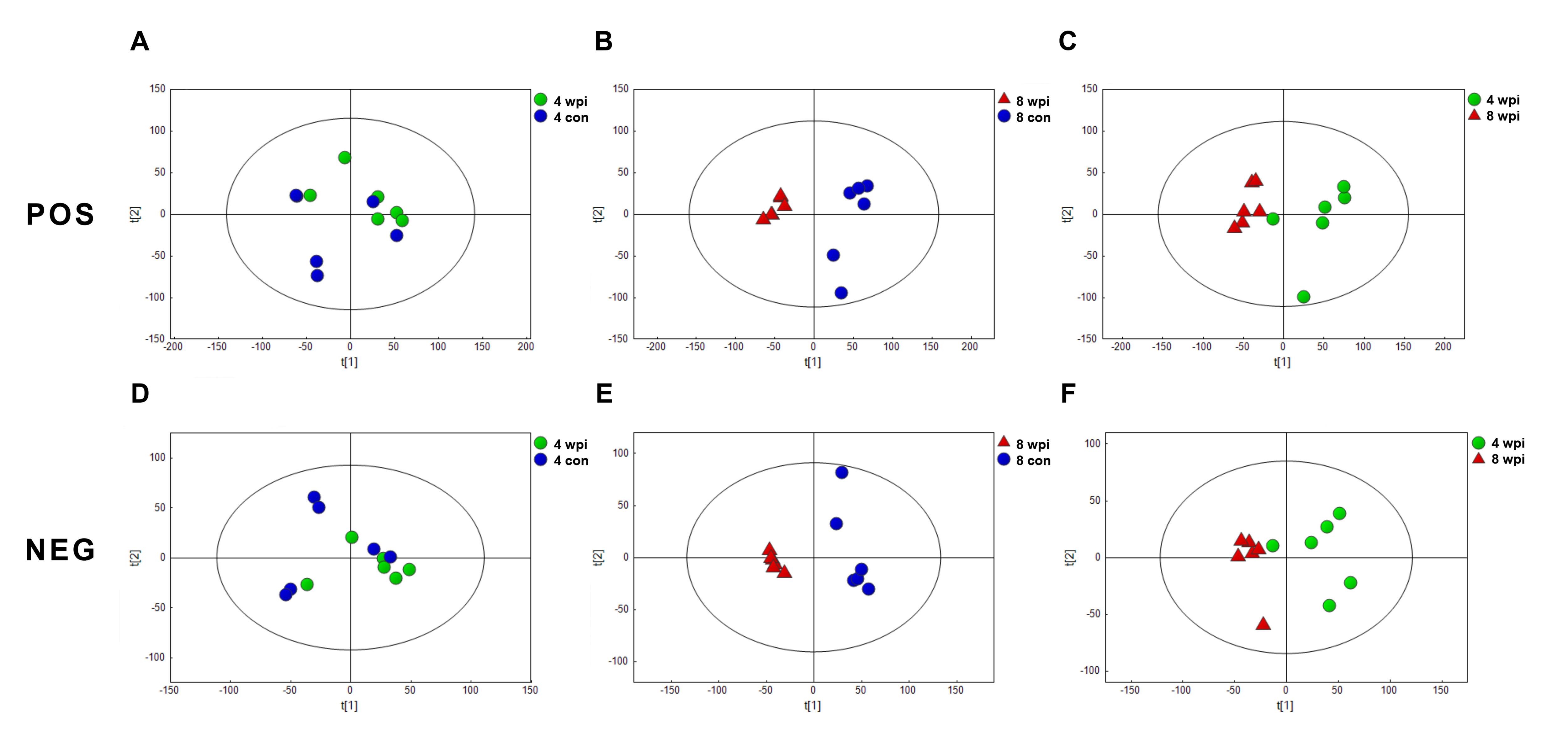

Supplement: Supplementary Figure 3 — PCA score scatter plots of metabolites obtained from UHPLC-QTOF-MS in ESI+ mode (A-C) and ESI− mode (D-F). Each color represents a group, and each point represents a sample. 4 wpi, 4 weeks post infection; 4 con, 4 weeks control; 8 wpi, 8 weeks post infection; 8 con, 8 weeks control. [file Image_3.tif]

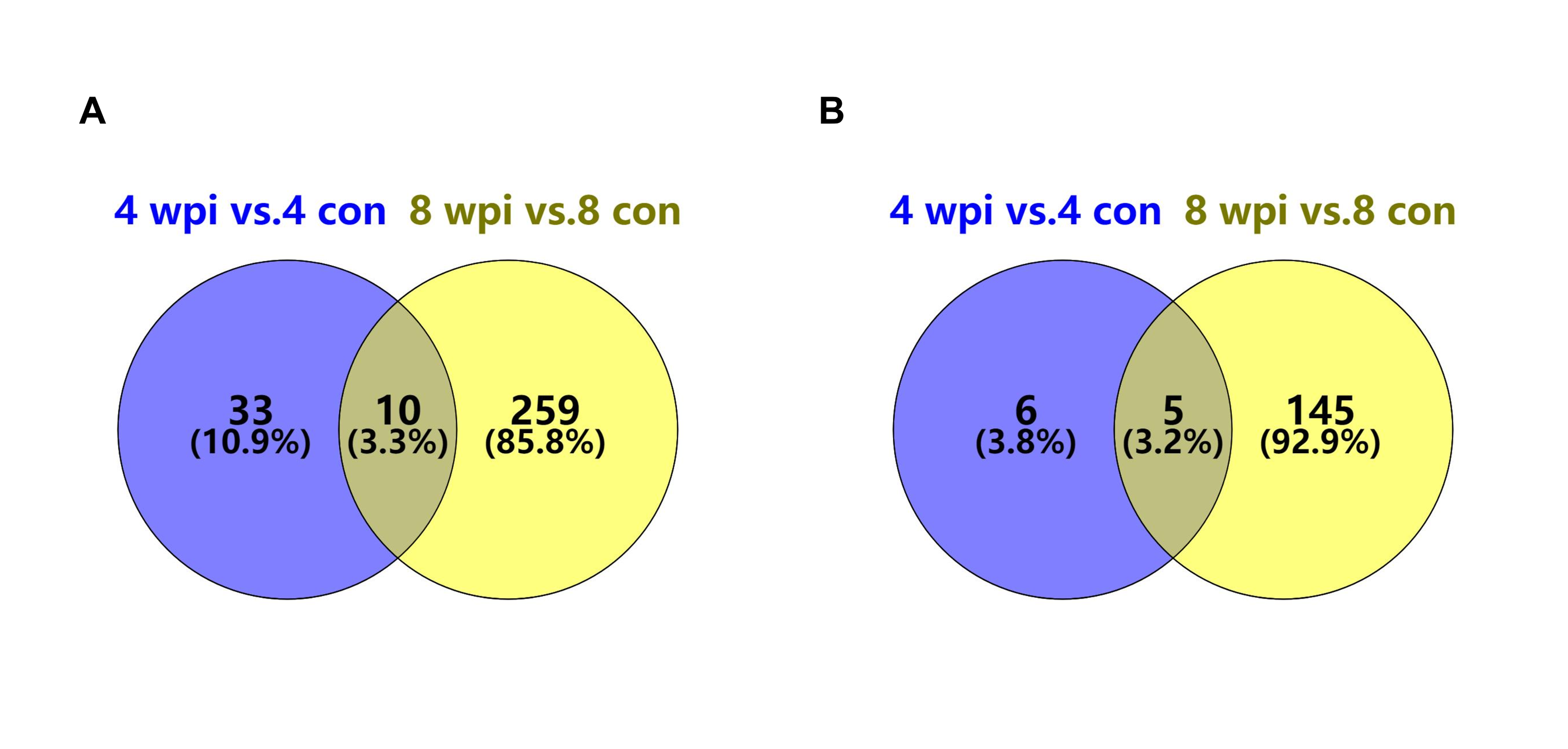

Supplement: Supplementary Figure 4 — Venn diagrams displaying (comparatively) the differentially expressed metabolites. Differential metabolites across comparison groups showing unique and common metabolites in ESI+ mode (A) and ESI− mode (B). [file Image_4.tif]
